# Osler and the Infected Letter

**DOI:** 10.3201/eid1105.040616

**Published:** 2005-05

**Authors:** Charles T. Ambrose

**Affiliations:** * University of Kentucky, Lexington, Kentucky, USA

**Keywords:** bioterrorism, Decontamination, Disinfection, Lazarettos, Osler, quarantine, smallpox, Variola, Viral stability, Mail

## Abstract

The spread of infectious agents through the mail has concerned public health officials for 5 centuries. The dissemination of anthrax spores in the US mail in 2001 was a recent example. In 1901, two medical journals reported outbreaks of smallpox presumably introduced by letters contaminated with variola viruses. The stability and infectivity of the smallpox virus are reviewed from both a historical (anecdotal) perspective and modern virologic studies. Bubonic plague was the contagious disease that led to quarantines as early as the 14th century in port cities in southern Europe. Later, smallpox, cholera, typhus, and yellow fever were recognized as also warranting quarantine measures. Initially, attempts were made to decontaminate all goods imported from pestilential areas, particularly mail. Disinfection of mail was largely abandoned in the early 20th century with newer knowledge about the spread and stability of these 5 infectious agents.

**Figure:**
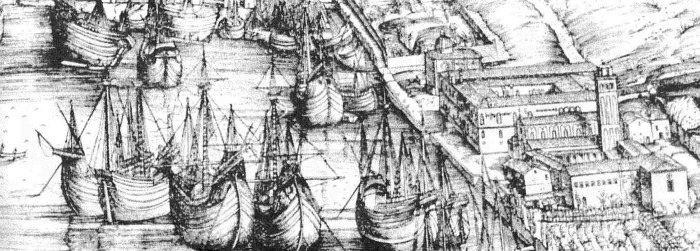
Ships docking at the *Lazzaretto Vecchio*, Venice, 14th century (1).

In January 1876, William Osler, a young Canadian physician, was recovering from a mild case of smallpox contracted while attending patients at Montreal General Hospital (Figure 1 [1,2]). In a letter written that same month to an old schoolmate (Arthus Jarvis), Osler described his illness and noted in closing, "You need not be afraid of this letter. I will disinfect it before sending" (2). Concern about disseminating smallpox through this letter was well founded. In his medical textbook of 1892, Osler would later write that smallpox can be conveyed by fomites: "the dried scales [of variola scabs] … as a dust-like powder … become attached to clothing and various articles …." (3).

**Figure 1:**
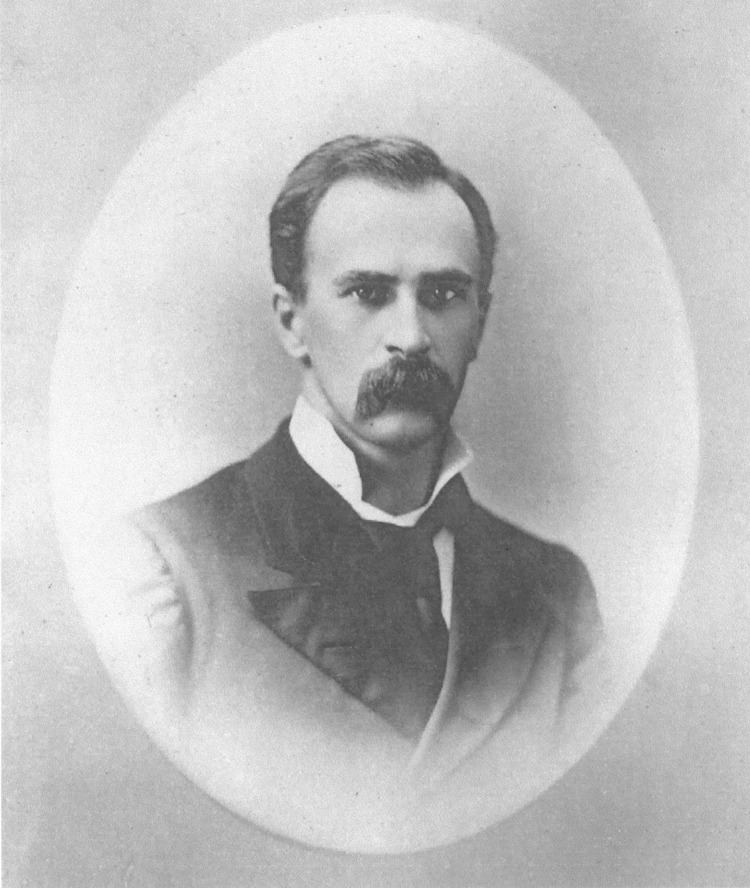
William Osler, age 28, 1877 (2).

## Stability of the Smallpox Virus

Long before Osler's time, the stability and infectivity of variola virus was well known, as illustrated by an example of germ warfare during the French and Indian War. In 1763, the British general Sir Jeffrey Amherst ordered that scab-ladened "Sundries" be delivered to the Ottawa Indians of Pennsylvania. Amherst hoped thereby to induce a debilitating smallpox epidemic among the Indians and conquer them (4).

Much later in the 1860s, a professional grave robber for the Medical College of Ohio in Cincinnati became incensed at tricks played on him by medical students. He delivered the corpse of a smallpox victim recently buried to the dissecting laboratory and intentionally infected many anatomy students (5).

The stability of the smallpox virus was often noted by 18th-century physicians in debates over the comparative merits of variolation and vaccination. Razzell cited a 1792 article describing how an English amateur inoculator dried smallpox scabs in peat smoke, stored them underground covered with camphor, and used them as long as 8 years later (6).

Razzell also reported the longest supposed survival of the variola virus, which caused an outbreak of smallpox in a town in Somerset in 1759. The coffin of a villager who had died of smallpox 30 years before was exhumed for transfer to a new grave site. The sexton accidentally put his spade through the oak coffin, which released "a most nauseous stench." The deceased was of such eminence that most of the villagers had attended the exhumation and reburial. "In a few days afterwards [sic], fourteen persons were seized with smallpox in one day" (6).

Today we would be skeptical of this story from 18th-century Somerset, but outbreaks of smallpox appeared occasionally in other isolated communities in England without any recognized living human source. For example, from 1908 to 1952, sporadic cases of variola appeared in Lancaster and Cheshire, textile mill counties. These outbreaks were likely introduced there by cotton imported from Egypt, where months before it had been contaminated with smallpox scabs (7).

Samples of the smallpox virus freeze-dried in a laboratory have been revived after storage for 20 years at Liverpool University (6). But long-term survival of this virus under natural conditions is another issue. In 1957, two London virologists using cell cultures examined smallpox scabs that had been stored in test tubes under ambient laboratory conditions. They found that the variola virus survived as long as 18 months (7). A comparable study from Leiden in 1968 used variola minor scabs collected and stored in 12 unsealed envelopes kept at room temperature. Each year the contents of a single envelope were cultured. The final envelope was examined 13 years later and, like the previous ones, showed virus particles still capable of replicating in culture (8).

The search for variola viruses surviving even longer was pursued in 1991 near Novosibirsk, Russia (9). "Bioweapons experts" searched for the variola virus in 19th-century smallpox victims mummified in the permafrost above the Arctic Circle. In the event of unusual thawing and flooding, the concern was that these corpses might become exposed and release infectious virus into the environment. In the 19th century, this region of Russia (Sakha Republic) was "ravaged by smallpox strains of extraordinary lethality" (9). Isolating and comparing them with preserved modern strains might identify genes contributing to virulence. To date, no live variola viruses have been isolated from Sakha. But the threat now is that "a sophisticated terrorist team might … go smallpox hunting on the permafrost" (9).

### Smallpox Transmitted in Letters

In 1876, Osler's concern was the danger his letter might pose to his friend Jarvis and not to cell cultures. Indeed, years later in 1901, articles in 2 respected medical journals incriminated letters as sources of 2 separate epidemics of smallpox (10,11).

The New York Medical Journal reported that smallpox had developed in a young lady in Saginaw, Michigan, after she received a letter from her sweetheart, a soldier in Alaska. He had written it while recovering from this disease. The infection subsequently spread to 33 other persons in Saginaw (10).

In addition, the April 1901 issue of the British Medical Journal reported an outbreak of 5 cases of smallpox at the Mormon headquarters in Nottingham, England, apparently after receipt of "letters or other fomites" from Salt Lake City, Utah, where smallpox was widespread (11). According to February 1901 issue of the Journal of the American Medical Association, 314 cases had been reported during the previous 3 months in Salt Lake City itself (12). The year before, the New York Times noted that Mormons opposed vaccination and had introduced a bill in the state legislature making it unlawful to compel vaccination (13).

### Other Fearsome Epidemics

In the fall of 2001, anthrax spores were sent in letters through the US mail. This event resulted in 18 confirmed cases of the disease, 5 deaths, and cross-contamination of perhaps 5,000 letters (14). Not since the fifth plague of Egypt, which may have been anthrax ("a very grievous murrain," Exodus 9), have people so panicked over the threat of this disease. However, in centuries past, many have fled from the sudden appearance of 5 other contagious diseases: smallpox, bubonic plague, yellow fever, typhus, and cholera. Malaria was such an expected seasonal affliction in many parts of the world that it was never perceived as an acute contagion.

When a smallpox epidemic struck Rome around 164 A.D., Galen is said to have hastily returned to his home in Pergamon on the Ionian Coast of modern-day Turkey. When plague returned to London in 1665, Thomas Sydenham, a physician, prudently sought safety in the countryside. In 1793, yellow fever swept through Philadelphia, then our federal capital. Alexander Hamilton left town, and President George Washington remained at Mount Vernon until the fall frost had been reported up North. In 1813, typhus decimated the French army in Moscow, forcing Napoleon to retreat to Paris. And in 1832, when cholera came to Kentucky, US Senator Henry Clay established a tent city on his estate outside Lexington for the 2,000 citizens who fled the town.

### Origin of Quarantines

In the 14th century, most citizens could not flee pestilences threatening their towns, but civil authorities sought to protect them by excluding suspected human carriers and merchandise from outside. Garrison gives a succinct history of early quarantines (15). When bubonic plague reached Europe in 1347, ports on the Mediterranean and Adriatic Sea were among the first to deny entry to ships coming from pestilential areas, notably from Turkey, the Middle East, or North Africa. Florence, on the Arno River, issued restrictions on travelers and goods as early as 1348. The Venetian Republic formally excluded "infected and suspected ships" in 1374 (15). The earliest such action in the Americans was by the Massachusetts Bay Colony in 1647 to 1648, when it barred ships coming from the West Indies thought to be carrying yellow fever (16).

The first official quarantine system is commonly ascribed to Ragusa (now called Dubrovnik), a port city located on the Dalmatian coast of the Adriatic Sea. There in 1377, and later when pestilences were abroad, incoming persons and ships were first isolated on a nearby island for 30 days (*trentina*) to await clinical signs of a contagion or evidence of continued good health. Detention of 40 days (*quarantina*) was instituted by the city of Marseille in 1383 and soon became the standard period of quarantine.

Later, other cities established isolation stations on shore or on nearby islands. Ragusa's use of an offshore island in 1377 was an early example of such a quarantine station. In spite of Ragusa's seeming priority, various sources claim that the first such station was a pest house built on the island of Sardinia in 1453 or buildings erected at Pisa near the church of San Lazzaro in 1464 (15,17). In North America during the 1743 epidemics of smallpox and yellow fever, an early quarantine station was established in Philadelphia on Providence Island in the Schuylkill River (18). Other major U.S. cities soon thereafter organized quarantine stations to cope with later epidemics of smallpox, yellow fever, typhus, and cholera.

### Lazarettos

**Figure 2:**
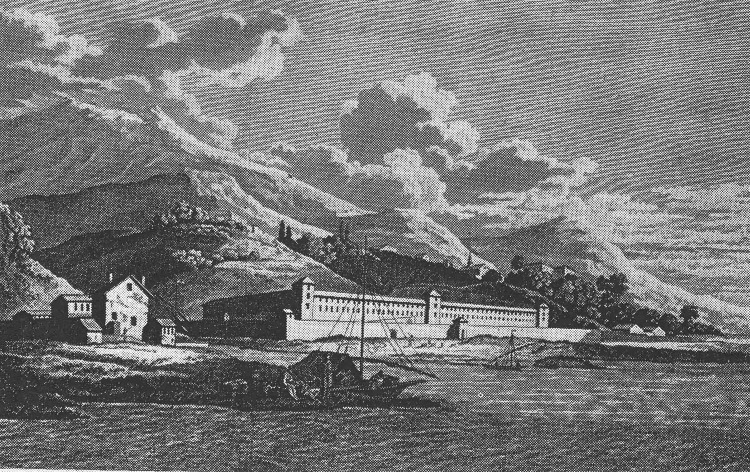
Lazaretto at Genoa, founded in 1467 (19).

Quarantine stations in southern Europe were originally called lazarettos. The origin of the term is uncertain. One 19th-century historian suggested that it is a corruption of the name of the church of Santa Maria di Nazaret, used as pest house in 15th-century Rome (18). But the Crusaders, who captured Jerusalem in 1099, had isolated and treated people with contagious diseases outside the city in the Hospital of St. Lazarus, the patron saint of lepers (17). In Venice (1403) quarantined ships were anchored at Lazzaretto Vecchio, an island in the lagoon. When the island acquired this particular name is not known. As noted above, in Pisa in 1464, persons were quarantined in a special building near the Church of San Lazzaro (15). An exhaustive, illustrated survey of lazarettos is given in John Howard's 1789 treatise on the subject (Figure 2) (19).

### Early Decontamination Measures

During the early Renaissance, clothing and other possessions of plague victims were often burned. In Italy and France during this period, the threat of plague compelled the destruction of great quantities of cloth prepared from cotton, wool, and silk recently imported from suspect countries. This precaution resulted in enormous economic loss and often in an immediate devastating local poverty (20).

The earliest attempts to decontaminate merchandise on ships coming from pestilential shores were made in Venice in the mid-1400s. Cargo was unloaded and fumigated with smoke from burning straw, pitch, tobacco, or even gunpowder. Cargo was also "perfumed." This term likely derived from burning fragrant herbs, juniper berries, aromatic gums (e.g., myrrh), and resinous wood in attempts to sterilize items. Early on, smoking sulfur was frequently employed, while in 18th-century Germany a mixture of sulfur, potassium nitrate (saltpeter), and wheaten bran (*Raucher Pulver*) was used (18).

### Disinfection of Mail

Karl F. Meyer, a physician-pathologist from Louisville, Kentucky, spent a lifetime researching the disinfection of mail (18). He determined that disinfection was first attempted in Venice around 1493 by dipping letters in vinegar. Later, other methods were used. By the early 1600s, decontamination of mail was practiced in much of Europe. In the United States in 1712, when yellow fever threatened Boston, mail from docking ships was first exposed to burning sulfur (17).

Very few letters from the early centuries of decontaminating mail are available today. But 1 rare specimen from 1485 does show evidence of having been dipped in vinegar (18). Since such treatment often rendered parts of a letter illegible, other less-damaging methods were employed, such as exposure to smoke and various fumes. The eventual widespread use of burning sulfur yielding sulfur dioxide (with its "sharp, irritating odor") may have been based on an ancient idea that the more foul a medicine, the more effective it might be.

In the late 19th century, sulfur gave way to chlorine or formaldehyde gas. In November 2001, chlorine dioxide gas was sprayed into the partly contaminated Hart Senate Office Building, while the Postal Service used a 10% solution of bleach to "sterilize" its mail sorting centers (21). Ion beam sterilization (high-energy electrons) and x-ray radiation have been considered for use on individual letters.

### Decontaminating Letters "Inside and Outside"

**Figure 3:**
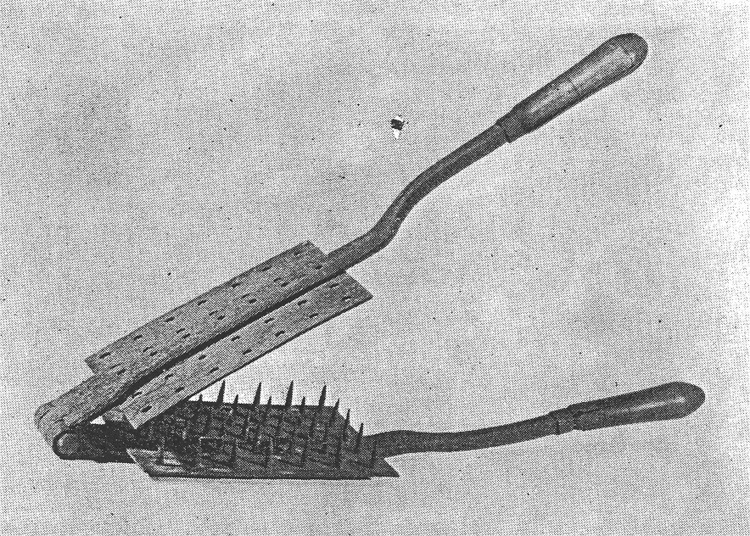
Rastel, a perforating mallet, ca. 1830 (18).

Sterilizing the outside of sealed envelopes did not ensure that the letter inside was safe. To allow penetration of sterilizing fumes or gases, initially envelopes were breached by cutting a small tip off one or more corners without exposing the content of the letter inside. In later years, multiple small holes were made in the envelope and its letter by means of a rastel, a hairbrush-size instrument consisting of 2 hinged metal plates (jaws), one of which held several rows of nails or small metal spikes (Figure 3 [18]). Clamping each letter between the jaws of the rastel produced several rows of small holes through the envelope and its contents, enabling gas to penetrate the interior (18).

When thousands of letters required fumigation, perforating each individually was not practical. Instead, they were laid out on screens, placed in an air-tight box (or in a boxcar; see figure on Table of Contents), and exposed to burning sulfur for ≈6 hours (18). Sterilizing the outside of envelopes protected the mail handlers, but the reader still remained at risk from the interior.

### Certification of Decontamination

**Figure 4:**
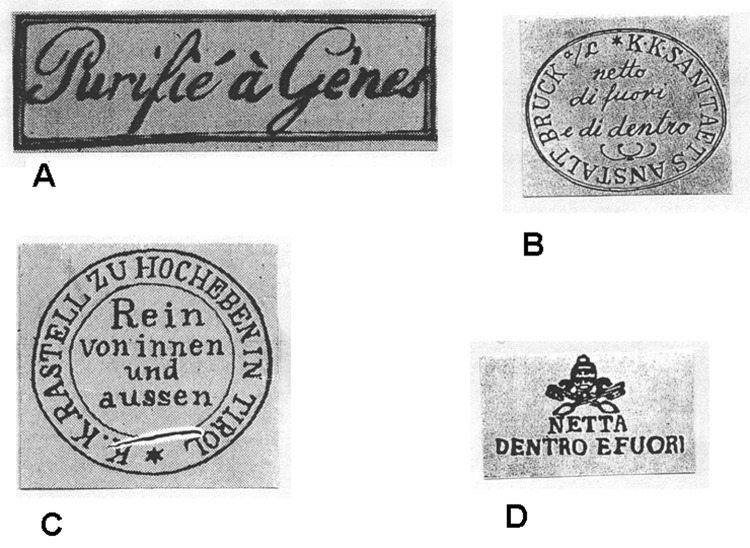
Various cachets from 19th-century envelopes. A) Genoa, 1813; B) Austria, 1830–1869; C) Vienna, 1831–1832; D) papal insignia (18).

Once letters had been decontaminated, some sort of certification had to be noted on them. As late as 1837, a paste or wax seal was affixed to fumigated letters. But this certification was impractical with large numbers of letters, so soon each letter was simply stamped, much like a modern-day postal cancellation. The following descriptions of these cancellation marks are taken from illustrations of some early 19th-century cachets (Figure 4 [18]).

A letter that had passed through Genoa in 1813 during the Napoleonic occupation bore the French stamp "Purifié à Gènes." Letters stamped at Leghorn in 1829 showed "LAZZERETTO SAN ROCCO DI LIVORNO." San Rocco was 1 of the 2 plague saints. During the early period when only the outside of envelopes were disinfected, an Italian cachet read "NETTA FUORAI E SPORCA DENTRO," or "clean outside and dirty inside." An 1830 cachet with the Papal insignia noted "NETTA DENTRO E FUORI" (clean inside and out). An 1831 stamp from Vienna read "Rein von innen und aussen" (clean within and out). A first-class letter from Jacksonville, Florida, in 1888 read simply "Fumigated" and thus did not define the extent of the procedure.

### Decline in Mail Disinfection

By the early 20th century, plague, typhus, and yellow fever were known to be transmitted by arthropod vectors, and cholera was known to be waterborne. Since letters seemed an unlikely means of spreading pestilences, disinfection of mail declined, but some authorities continued to see a potential risk in mail from patients with tuberculosis and leprosy. Meyer noted that as late as 1953, letters leaving a German tuberculosis sanatorium were first fumigated with formaldehyde fumes. Likewise in the United States, as late as 1968 mail leaving the leprosarium at Carville, Louisiana, was first sterilized by baking in electric ovens (18).

### Conclusions about Osler's Letter

Osler did not say how he would disinfect his 1876 letter to Jarvis. The letter shows no vinegar stains. The sterilizing value of dry heat (oven) and moist heat (autoclave) was not established until 1881 by Koch and others (22). The causative agent of smallpox was not visualized microscopically until 1887, when Buist first observed small clumps of virus particles now called Guarnieri bodies in infected tissues. The variola virus was first cultivated in 1935 by Torres and Teixeria on the chorioallantoic membrane of embryonated eggs (23).

Being a pathologist, Osler may have used formaldehyde vapors to sterilize his letter. In any case, we do not hear that Jarvis ever contracted smallpox from it. Indeed, correspondence between Osler and Jarvis continued at least through 1910 (24). Osler died in 1919 and Jarvis in 1936 (L. Russell, pers. comm.).
