# Correction: Vol. 11, No. 5

**DOI:** 10.3201/eid1106.C21106

**Published:** 2005-06

**Keywords:** Correction, errata, erratum

In “Osler and the Infected Letter,” by Charles T. Ambrose, an error occurred. Yellow fever swept through Philadelphia in 1793.

The corrected article appears online at http://wwwnc.cdc.gov/eid/article/11/5/04-0616_article.htm.

We regret any confusion these errors may have caused.

